# Preclinical Studies of Chemotherapy Using Histone Deacetylase Inhibitors in Endometrial Cancer

**DOI:** 10.1155/2010/923824

**Published:** 2010-02-04

**Authors:** Noriyuki Takai, Hisashi Narahara

**Affiliations:** Department of Obstetrics and Gynecology, Faculty of Medicine, Oita University, Hasama-machi, Yufu-shi, Oita 879-5593, Japan

## Abstract

Because epigenetic alterations are believed to be involved in the repression of tumor suppressor genes and promotion of tumorigenesis in endometrial cancers, novel compounds endowed with a histone deacetylase (HDAC) inhibitory activity are an attractive therapeutic approach. In this review, we discuss the biologic and therapeutic effects of HDAC inhibitors (HDACIs) in treating endometrial cancer. HDACIs were able to mediate inhibition of cell growth, cell cycle arrest, apoptosis, and the expression of genes related to the malignant phenotype in a variety of endometrial cancer cell lines. Furthermore, HDACIs were able to induce the accumulation of acetylated histones in the chromatin of the p21^WAF1^ gene in human endometrial carcinoma cells. In xenograft models, some HDACIs have demonstrated antitumor activity with only few side effects. In this review, we discuss the biologic and therapeutic effects of HDACIs in treating endometrial cancer, with a special focus on preclinical studies.

## 1. Introduction

Endometrial cancer is the seventh most common malignancy among women worldwide. Despite the fact that most cases are diagnosed at an early stage, the death rate has increased steadily over the past 20 years. The lack of an effective, standardized adjuvant treatment for women at a high risk of recurrence has contributed to these disappointing results (reviewed in [1]). The most frequent genetic alteration in type I endometrioid carcinoma is PTEN inactivation by mutation, followed by microsatellite instability and mutations of K-ras and *β*-catenin. In type II cancers, p53 mutation is the most frequent genetic alteration, followed by inactivation of p16 and e-cadherin and amplification of Her2/neu (reviewed in [1]).

One of the most important mechanisms in chromatin remodeling is the post-translational modification of the N-terminal tails of histones by acetylation, which contributes to a “histone code” determining the activity of target genes [2]. Transcriptionally silent chromatin is composed of nucleosomes in which the histones have low levels of acetylation on the lysine residues of their amino-terminal tails. Acetylation of histone proteins neutralizes the positive charge on lysine residues and disrupts the nucleosome structure, allowing unfolding of the associated DNA with subsequent access by transcription factors, resulting in changes in gene expression. Acetylation of core nucleosomal histones is regulated by the opposing activities of histone acetyltransferases (HATs) and histone deacetylases (HDACs). HDACs catalyze the removal of acetyl groups on the amino-terminal lysine residues of core nucleosomal histones, and this activity is generally associated with transcriptional repression. HDACs remove the acetyl groups which then induce a positive charge on the histones, and this suppresses gene transcription, including tumor suppressor genes silenced in cancer. Moreover, acetylation of histones facilitates destabilization of DNA-nucleosome interaction and renders DNA more accessible to transcription factors [3]. Aberrant recruitment of HDAC activity has been associated with the development of certain human cancers [4]. HDAC inhibitors (HDACIs) can inhibit cancer cell growth in vitro and in vivo, revert oncogene-transformed cell morphology, induce apoptosis, and enhance cell differentiation.

The classes of HDACIs that have been identified are: (a) organic hydroxamic acids (e.g., Trichostatin A (TSA) and suberoylanilide bishydroxamine (SAHA)) (b) short-chain fatty acids (e.g., butyrates and valproic acid (VPA)), (c) benzamides (e.g., MS-275), (d) cyclic tetrapeptides (e.g., trapoxin), and (e) sulfonamide anilides [5] (see Table 1).

In this review, we discuss the biologic and therapeutic effects of HDACIs in treating endometrial cancer, with a special focus on preclinical studies.

## 2. Mechanism of Action

Histone deacetylases (HDACs) comprise a family of 18 genes that are subdivided into four classes [6]. Classes I, II, and IV are referred to as ‘‘classical” HDACs and are generally simultaneously targeted by most HDACIs (Table 1). HDACIs were initially discovered on the basis of their ability to reverse the malignant phenotype of transformed cells in culture. It has been shown that HDACIs carry the potential to activate differentiation programs on one hand, while on the other hand they were also shown to inhibit cell proliferation by cell cycle arrest in the G1 and/or G2 phases of the cell cycle and to induce apoptosis in cultured transformed cells. p21^WAF1^ and p27^KIP1^ are cyclin-dependent kinase inhibitors (CDKIs) that bind to cyclin-dependent kinase complexes and decrease kinase activity, and may act as key regulators of the G0/G1 accumulation (reviewed in [7]). The p21^WAF1^ expression in particular is induced by HDACIs in various cell lines. Additionally, this event is associated with both an increase in histone acetylation in the promoter region of the p21^WAF1^ gene and a selective loss of a specific HDAC enzyme, HDAC1, in the same region [8]. Therefore, the upregulation of p21^WAF1^ is a direct consequence of HDACIs on p21^WAF1^ transcription. In the future, testing should be conducted using p21^WAF1^-negative cell lines to see if p21^WAF1^ is absolutely required for HDACI-induced growth arrest. Takai et al. examined the effect of HDACIs on the expression of p21^WAF1^ and p27^KIP1^ in endometrial cancer cells by Western blot analysis. HDACIs markedly upregulated the level of p21^WAF1^ and p27^KIP1^ proteins, which were expressed at negligible levels in the untreated cell lines. Conversely, HDACIs decreased the levels of cyclin D1 and cyclin D2. HDACIs decreased the bcl-2 levels. E-cadherin binds to *β*-catenin and can act as a tumor suppressor gene; its promoter has CpG islands which are frequently methylated in selected cancers. HDACIs markedly increased the expression level of E-cadherin in endometrial cancer cells and exhibited antiproliferative activity in these cells [9–14]. HDACIs have also been shown to generate reactive oxygen species (ROS) in solid tumor and leukemia cells [15–17], which may contribute to the mechanism. HDACIs have been shown to inhibit angiogenesis. HDACIs repress the expression of proangiogenic factors such as HIF1*α*, VEGF, VEGF receptor, endothelial nitric oxide synthase, IL-2 and IL-8 and the induction of antiangiogenic factors, such as p53 and von Hippel-Lindau (reviewed in [18]). HDACIs should not be considered to act solely as enzyme inhibitors of HDACs. A large variety of nonhistone transcription factors and transcriptional co-regulators are known to be modified by acetylation. HDACIs can alter the degree of acetylation nonhistone effector molecules and thereby increase or repress the transcription of genes by this mechanism. Examples include: ACTR, cMyb, E2F1, EKLF, FEN 1, GATA, HNF-4, HSP90, Ku70, NF*κ*B, PCNA, p53, RB, Runx, SF1 Sp3, STAT, TFIIE, TCF, YY1, and so forth, (reviewed in [18]).

## 3. Overview and Preclinical Studies of HDACIs

A variety of structurally distinct classes of compounds that inhibit deacetylation of both histone and non-histone proteins have gradually been identified (Table 1). Despite the shared capacity of each class of HDACIs to promote histone acetylation, individual HDACIs exert different actions on signal transduction and the induction of differentiation and/or apoptosis. Table 2 shows data from different reports investigating endometrial cancer cell lines treated with different classes of HDACIs. Many of the in vitro studies use the Ishikawa cell line. Nishida. succeeded in establishing of a well-differentiated endometrial adenocarcinoma cell line, Ishikawa cells, from a 39-year old Japanese patient more than 20 years ago [19]. Because this cell line bears estrogen and progesterone receptors, the cells have been used in numerous basic research areas such as reproductive biology and molecular science.

### 3.1. Trichostatin A (TSA)

The trichostatins were initially isolated from Streptomyces hygroscopicus as antifungal antibiotics in 1976 [20, 21]. About 10 years later, TSA and its analogues were discovered to induce cell differentiation of murine erythroleukemia cells and to induce hyperacetylation of histone proteins at nanomolar concentrations. TSA has been extensively studied; it has antitumor activity and can induce differentiation of some cancer cell lines, but its clinical utility has been restricted because of toxic side-effects in vivo [22]. TSA causes mitotic arrest through the formation of aberrant mitotic spindles, probably by interfering with chromosome attachment, but does not affect mitotic microtubules [22]. This effect may account for the higher cytotoxicity of TSA in comparison to other HDACIs (i.e., suberoylanilide bishydroxamine). HDAC inhibition is not believed to have a generalized effect on the genome, but only on the transcription of a small subset of the genome. Differential display analysis of transformed lymphoid cell lines revealed that the expression of only 2%–5% of transcribed genes is changed significantly after treatment with TSA [23]. The effective dose of TSA that inhibited 50% clonal growth (ED50) of the endometrial cancer cell lines (Ishikawa, HEC-1B, HEC59, RL95-2, KLE, and AN3CA) was calculated, and ranged between 5.1 × 10^−8^ M and 1.9 × 10^−7^ M [9] (Table 2). Dowdy et al. demonstrated that combined treatment with TSA and paclitaxel caused synergistic inhibition of cell growth of Ark2 and KLE endometrial cancer cells [24]. These effects were confirmed in a mouse xenograft model. Treatment with TSA and paclitaxel led to a significant increase in acetylated tubulin and microtubule stabilization. This study provides the evidence of nonhistone protein acetylation as one possible mechanism by which HDACIs reduce cancer growth.

### 3.2. Suberoylanilide Bishydroxamine (SAHA, Vorinostat)

Hydroxamic acid type inhibitors make up the largest and broadest group of HDACIs described to date. The inhibition of HDACs by SAHA occurs through a direct interaction with the catalytic site of the enzyme, as shown by X-ray crystallography studies [25]. Among the synthetic HDACIs, SAHA is the most advanced candidate as a cancer therapeutic drug, and is under phase I and II clinical trials [26, 27]. SAHA has significant antitumor activity against many tumor types at dosages that can be tolerated by patients when administered intravenally and orally [28]. Some HDACIs (e.g., TSA and trapoxin) are of limited therapeutic use due to poor bioavailability in vivo and have toxic side effects at high doses. SAHA, however, is relatively safe and non-toxic in vivo. The effective dose of SAHA that inhibited 50% clonal growth (ED50) of the endometrial cancer cell lines (Ishikawa, HEC-1B, HEC59, RL95-2, KLE, and AN3CA) was calculated and ranged between 7.8 × 10^−7^ M and 3.1 × 10^−6^ M [9] (Table 2).

### 3.3. m-carboxycinnamic Acid Bishydroxamide (CBHA)

CBHA is a member of a recently synthesized family of hybrid polar compounds that have been shown to be inhibitors of HDAC [29] and potent inducers of transformed cell growth arrest and terminal differentiation at micromolar (LD50 range, 1–4 *μ*M) concentrations [30]. The effective dose of CBHA that inhibited 50% clonal growth (ED50) of the endometrial cancer cell lines (Ishikawa, HEC-1B, HHUA) was calculated and ranged between 1.8 × 10^−6^ M and 2.5 × 10^−6^ M for CBHA [10] (Table 2). On the other hand, normal endometrial epithelial cells were viable after treatment with the same doses of CBHA that induced growth inhibition of endometrial cancer cells [10].

### 3.4. Scriptaid

Using a high-throughput system based on a stably integrated transcriptional reporter to screen a library of 16,320 compounds (DIVERset, Chembridge, San Diego, CA), Su et al. identified a novel HDACI, termed Scriptaid [31]. Nullscript, which possesses a shorter side-chain (3C) than Scriptaid (5C) between the tricyclic core and the carbonyl group, was inactive in transcriptional facilitation. This confirms that the linker chain has to be a certain length for HDAC inhibition to occur. Scriptaid has a common structure with TSA and SAHA, that is, a hydroxamic acid zinc-binding group linked via a spacer (5 or 6 CH2) to a hydrophobic group [31]. Using an immunoblotting assay of histone deacetylation, Su et al. demonstrated that Scriptaid is a potent HDACI with a >100-fold increase in histone acetylation, with relatively low toxicity [31]. Scriptaid conferred the greatest effect on augmentation of the signal transduction TGF*β* pathway, including a number of human suppressor genes such as SMAD4 [31]. The effect of Scriptaid in human cancers, however, has not been fully examined. A recent study by Keen et al. indicated that Scriptaid had a significant growth-suppressing effect on ER-negative human breast cancer cells [32]. The effective dose of Scriptaid that inhibited 50% clonal growth (ED50) of the Ishikawa endometrial cancer cell line was calculated at 9.0 × 10^−6^ M [11] (Table 2). On the other hand, normal endometrial epithelial cells were viable after treatment with the same doses of Scriptaid that induced growth inhibition of endometrial cancer cells [11].

### 3.5. Sodium Butyrate (NaB)

It was first reported in 1978 that millimolar concentrations of sodium butyrate (NaB) inhibited HDACs in vitro [33]. NaB is normally present in the human colon as a product of the metabolic degradation of complex carbohydrates by colonic bacteria and regulates the physiological differentiation of colonocytes, suggesting its possible use in the prevention of colorectal cancer and the treatment of premalignant and neoplastic lesions. Butyrate and its derivatives have a long history of safe clinical use in the treatment of inherited and acquired metabolic disorders. Some studies suggest that many of the cellular activities of phenylbutyrate are more dependent on its butyric acid component than its phenyl group. A recent study by Terao et al. indicated that NaB had a significant growth-suppressing effect on human endometrial and ovarian cancer cells irrespective of their p53 gene status [34]. NaB, a low-potency HDACI, has been extensively studied; it has antitumor activity and can induce differentiation of some cancer cell lines, but its clinical utility has been restricted by its short half-life (5 minutes), limiting the ability to achieve a therapeutic plasma level. NaB and phenylbutyrate are degraded rapidly after i.v. administration and therefore require high doses exceeding 400 mg/kg/day [35]. Furthermore, these compounds are not specific for HDACs as they also inhibit phosphorylation and methylation of proteins as well as DNA methylation [35]. The effective dose of NaB that inhibited 50% clonal growth (ED50) of the endometrial cancer cell lines (Ishikawa, HEC-1B, HEC59, RL95-2, KLE, and AN3CA) has been calculated and ranged between 8.3 × 10^−4^ M and 4.1 × 10^−3^ M for NaB [9] (Table 2).

### 3.6. Valproic Acid (VPA)

Valproic acid, a shortchain fatty acid, has been approved for clinical use in the treatment of epilepsy and is frequently used in clinical trials and for in vitro research based on its HDAC inhibitory effect at comparatively high (millimolar) concentrations [36]. Valproic acid has also been identified as an antiproliferative agent and HDACI [37]. Some HDACIs (e.g., TSA and trapoxin) are of limited therapeutic use due to poor bioavailability in vivo as well as toxic side effects at high doses, but VPA is relatively safe and non-toxic in vivo. The effective dose of VPA that inhibited 50% clonal growth (ED50) of the endometrial cancer cell lines (Ishikawa, HEC-1B, HEC59, RL95-2, KLE, and AN3CA) has been calculated and ranged between 7.0 × 10^−4^ M and 3.8 × 10^−3^ M [9] (Table 2).

A previous study tested the ability of VPA to inhibit the growth of human HEC-1B endometrial cancer tumors growing in immunodeficient mice [9]. Administration of VPA remarkably suppressed the growth of the tumors (*P* <.01). No significant differences in either the mean weights, histology of the internal organs, and mean blood chemistries, including liver parameters and hematopoietic values, were found between diluent-treated mice and those that received 5 weeks of therapy. These tumors were sampled for the expression of p21^WAF1^ using immunohistochemistry on formalin-fixed paraffin-embedded sections. HEC-1B endometrial cancer cells treated with VPA showed strong nuclear staining. Control cancer cells from untreated mice had negative or focal weak staining for p21^WAF1^. This in vivo study shows that VPA at 0.3–1.5 mM inhibited cell proliferation, induced cell cycle arrest and stimulated apoptosis in endometrial cancer cells. This range of concentrations of VPA can be achieved in a patient's serum when that patient is receiving a daily dose of 20–30 mg/kg for epilepsy. These data are also consistent with the in vitro data. This anticancer activity occurred without any major side-effects, raising hopes that VPA may become a useful therapy for endometrial cancers. Furthermore, VPA has convenient pharmacokinetic properties with a significantly longer biological half-life than the other HDACIs [38].

### 3.7. MS-275 (Entinostat)

MS-275 (MS-27-275) is a synthetic novel benzamide which exerts HDAC inhibitory activity and also induces the expression of the cyclin-dependent kinase inhibitor p21^WAF1^ and gelsolin, and changes the cell cycle distribution [38, 39]. MS-275 has shown antiproliferative activity in various in vitro and in vivo human tumor models [40, 41], and is currently being tested in clinical trials involving patients with solid tumors or hematological malignancies [42]. The effective dose of MS-275 that inhibited 50% clonal growth (ED50) of the endometrial cancer cell lines (Ishikawa, HHUA, HEC-1B and RL95-2) was calculated and ranged between 7.8 × 10^−7^ M and 2.2 × 10^−6^ M [12] (Table 2). On the other hand, normal endometrial epithelial cells were viable after the treatment with the same doses of MS-275 that induced growth inhibition of endometrial cancer cells [12]. Jiang et al. reported that over the course of 4 days, there was a 60% reduction in the serous endometrial cancer cell line Ark2 cell counts by MS-275 (which they called HDAC-I1) (0.5 *μ*M) treatments, as compared to controls treated with DMSO solvent (Table 2). They reported growth inhibition of both endometrioid (Ishikawa and AN3) and serous (Ark2) endometrial carcinomas [43].

### 3.8. M344

Synthetic amide analogs were discovered to have a common structure with TSA [44]. Using an in vitro enzyme inhibition assay of histone deacetylation, Jung et al. demonstrated that M344 is a potent HDACI and an inducer of terminal cell differentiation [44]. The effective dose of M344 that inhibited 50% clonal growth (ED50) of the Ishikawa endometrial cancer cell line was calculated at 2.3 × 10^−6^ M [13] (Table 2). On the other hand, normal endometrial epithelial cells were viable after the treatment with the same doses of M344 that induced growth inhibition of endometrial cancer cells [13].

### 3.9. Apicidin

Cyclic peptide HDACIs can be further divided into two classes: those with an epoxyketone group such as HC-toxin and trapoxin, and those without such a group (apicidin, depsipeptide or FK228). Apicidin is a novel cyclic tetrapeptide with a potent broad spectrum of antiprotozoal activity against Apicomplexan parasites [45]. Its structure is related to trapoxin, a potent HDACI, and some biological activity, including antiproliferative and toxic effects, have been shown in some cancer cell lines [46]. The effective dose of apicidin that inhibited 50% clonal growth (ED50) of the Ishikawa endometrial cancer cell lines was calculated at 1.0 × 10^−6^ M [14] (Table 2). On the other hand, normal endometrial epithelial cells were viable after the treatment with the same doses of HDACIs that induced growth inhibition of endometrial cancer cells [14]. Ueda et al. [14] and Ahn et al. [47] independently demonstrated that apicidin has antitumor properties on Ishikawa endometrial cancer cells by selectively inducing the genes related to cell cycle arrest and apoptosis.

### 3.10. Psammaplin A

Psammaplin A (PsA) is a natural bromotyrosine derivative from a two-sponge association, *Poecillastra sp*. and *Jaspis sp.*, which was first isolated from the *Psammaplysilla* sponge [45]. It was reported that PsA has antibacterial and antitumor properties, and also inhibits various enzymes including topoisomerase II, farnesyl protein transferase, leucine amino peptidase, and chitinase (reviewed in [48]). Recently, it was reported that PsA inhibits both HDAC and DNA methyltransferase (DNMT) as epigenetic modifiers of the tumor suppressor gene [49]. PsA caused antiproliferative activity and induced cell cycle arrest or apoptosis in Ishikawa human endometrial cancer cells. PsA inhibited the proliferation of Ishikawa cells in a dose-dependent manner. The 50% inhibitory concentration (IC50) of PsA was found to be 5 *μ*g/mL (7.5 × 10^−6^ M) after 48 h treatment (Table 2). PsA increased the proportion in the G1 phase and G2/M phases of the cell cycle [48].

### 3.11. Oxamflatin

Oxamflatin is an aromatic sulfonamide derivative with a hydroxamic acid group that was identified as a compound inducing the morphological reversion of v-K-*ras*-transformed NIH3T3 cells from a chemical library [50]. In addition, the morphology of NIH3T3 cells transformed by various other oncogenes such as v-*sis*, v-*src*, MEK^EE^ or v-*fos* was also reverted by oxamflatin. Kim et al. analysed the effect of oxamflatin on the proliferation of eight mouse and human tumor cell lines. The 50% inhibitory concentrations of oxamflatin for all the cell lines except CCD-19Lu, a normal human lung cell line, were below 0.72 *μ*g/mL, while that for CCD-19Lu was 1.4 *μ*g/mL [51]. Over the course of 4 days, there was a 78% reduction in the serous endometrial cancer cell line Ark2 cell counts by oxamflatin (0.25 *μ*M) treatments, as compared to controls treated with DMSO solvent. The most striking observation is the 95% reduction in cell counts following the administration of 0.75 *μ*M oxamflatin to Ark2 cells [43]. This report resulted in growth inhibition of both endometrioid (Ishikawa and AN3) and serous (Ark2) endometrial carcinomas.

## 4. Conclusions

In this review, we summarize recent preclinical studies on the use of HDACIs, especially in human endometrial cancer cells. Many questions are currently still unanswered with respect to HDACI specificities for definite tumor subtypes and the molecular mechanisms underlying HDACI-induced differentiation, cell cycle arrest and apoptosis. In addition, the regulation mechanisms of the specific gene expression and recruitment of HDAC complex to the specific promoter sites remain still to be determined. Also, it is still unclear to what extent different HDACs exhibit different and potentially overlapping functions, and it is important to distinguish the HDAC specificity of HDACIs for the development of selective therapy on the molecular level. Further work is needed to improve our understanding of why transformed cells are more susceptible to the effect of HDACIs than normal cells. Also, combinations of HDACIs with differentiation-inducing agents, with cytotoxic agents, and even with gene therapy may represent novel therapeutic strategies and new hope for the treatment of endometrial cancer.

## Figures and Tables

**Table 1:** Overview of frequently used histone deacetylase inhibitors that are available for clinical and research purposes.

Substance groups	Derivatives	Isotype
Hydroxamates	Trichostatin A (TSA)	I, II
	Suberoylanilide hydroxamic acid (SAHA, vorinostat)	I, II, IV
	LBH589 (panobinostat)	I, II, IV
	PCI24781 (CRA-024781)	I, IIb
	LAQ824	I, II
	PXD101 (belinostat)	I, II, IV
	ITF2357	I, II
	SB939	Unknown
	JNJ-16241199 (R306465)	I
	m-carboxycinnamic acid bishydroxamide (CBHA)	
	Scriptaid	
	Oxamflatin	
	Pyroxamide	
	Cyclic hydroxamic acid containing peptides (CHAPs)	

Short chain fatty acids	Butyrate	I, IIa
	Valproate	I, IIa
	AN-9	
	OSU-HDAC42	

Benzamides	MS-275 (entinostat)	1, 2, 3, 9
	MGCD0103	1, 2, 3, 11
	Pimelic diphenylamide	1, 2, 3
	M344	
	N-acetyldinaline (CI-994)	

Cyclic tetrapeptides	Apicidine	I, II
	Trapoxins	
	HC-toxin	
	Chlamydocin	
	Depsipeptide (FR901228 or FK228) (romidepsin)	1, 2, 4, 6

Sulfonamide anilides	N-2-aminophenyl-3-[4-(4-methylbenzenesulfonylamino)-phenyl]-2-propenamide	

Others	Depudecin	
	NDH-51	
	KD5150	Pan-HDACI

Class I: HDAC1, -2, -3 and -8; class IIa: HDAC4, -5, -7, and -9; class IIb: HDAC 6, and -10; class III: SIRT1-7; class IV: HDAC11.

**Table 2:** Data investigating endometrial cancer cell lines treated with different classes of HDACIs.

HDACI	Cell line	ED50 (M)
TSA	Ishikawa	5.2 × 10^−8^
	HEC-1B	5.1 × 10^−8^
	HEC59	7.0 × 10^−8^
	RL95-2	9.8 ×10^−8^
	KLE	7.2 ×10^−8^
	AN3CA	1.9 ×10^−8^
	Ark2	2.5 ×10^−8^

SAHA	Ishikawa	7.8 × 10^−7^
	HEC-1B	7.8 × 10^−7^
	HEC59	1.2 × 10^−6^
	RL95-2	2.4 ×10^−6^
	KLE	2.5 ×10^−6^
	AN3CA	3.1 ×10^−6^

CBHA	Ishikawa	1.8 × 10^−6^
	HHUA	2.5 × 10^−6^
	HEC-1B	2.2 × 10^−6^

Scriptaid	Ishikawa	9.0 × 10^−6^

NaB	Ishikawa	8.3 × 10^−4^
	HEC-1B	8.4 × 10^−4^
	HEC59	1.8 × 10^−3^
	RL95-2	3.0 ×10^−3^
	KLE	3.9 ×10^−3^
	AN3CA	4.1×10^−3^

VPA	Ishikawa	7.0 × 10^−4^
	HEC-1B	7.5 × 10^−4^
	HEC59	8.2 × 10^−4^
	RL95-2	2.5 ×10^−3^
	KLE	2.3 ×10^−3^
	AN3CA	3.8 ×10^−3^

MS-275	Ishikawa	9.7 × 10^−7^
	HEC-1B	2.2 × 10^−6^
	RL95-2	1.0 × 10^−6^
	HHUA	7.8 ×10^−7^
	AN3CA	5.0 × 10^−7^
	Ark2	5.0 ×10^−7^

M344	Ishikawa	2.3 × 10^−6^

Apicidine	Ishikawa	1.0 × 10^−6^

PsA	Ishikawa	7.5 × 10^−6^

Oxamflatin	Ishikawa	2.5 × 10^−7^
	AN3CA	2.5 × 10^−7^
	Ark2	2.5 × 10^−7^
